# Real-world incidence and prevalence of systemic lupus erythematosus in Alberta, Canada

**DOI:** 10.1007/s00296-018-4091-4

**Published:** 2018-07-09

**Authors:** Francis Fatoye, Tadesse Gebrye, Lawrence W. Svenson

**Affiliations:** 1 Department of Health Professions, Faculty of Health, Psychology, and Social Care, Manchester Metropolitan University, Brooks Building, 53 Bonsall Street, Manchester, M15 6GX UK; 2 Analytics and Performance Reporting Branch, Alberta Health, Edmonton, Canada; 3 Division of Preventive Medicine, University of Alberta, Edmonton, Canada; 4 School of Public Health, University of Alberta, Edmonton, Canada; 5 Department of Community Health Sciences, University of Calgary, Calgary, Canada

**Keywords:** Systemic lupus erythematosus, Prevalence, Incidence, Epidemiology, Alberta

## Abstract

Systemic lupus erythematosus (SLE) is rather uncommon than rare. The purpose of this study was to estimate the incidence and prevalence of SLE in the population of Alberta, Canada, using administrative health data. Multiple population-based data sources, including the Alberta Health Care Insurance Plan Central Stakeholder Registry (AHCIP CSR), Fee-For-Service, and Hospital Discharge Abstract Database were used. Age- and sex-specific incidence and prevalence rates, and 95% confidence intervals (CI), were computed using the AHCIP CSR mid-year population estimates as the denominator, for the period of 2000–2015. The overall incidence of SLE for all age groups was 4.43 (95% CI 3.65, 5.04) per 100,000 population. The overall incidence in male and female of all age groups was 1.26 (95% CI 0.72, 1.76) and 7.69 (95% CI 6.22, 8.81) per 100,000 population, respectively. A prevalence of 47.99 per 100,000 (male = 13.5, female = 83.2) of SLE was observed for the year 2000 and has increased to 90 (male = 25.5, female = 156.7) per 100,000 population in 2015. Over the 16-year period, the incidence of SLE in women was approximately six times higher than in men (odds ratio = 6.16). The highest and lowest incidence was recorded in 2001 and 2015, respectively. Despite the stable incidence of SLE, the findings of the study confirms that the prevalence of SLE has increased over the 16-year period. The increase in prevalence of SLE in Alberta will have an impact on health service utilizations. This finding can be used for planning and evaluating health services for this group of patients. Further studies are required to determine the economic burden of the condition.

## Introduction

Systemic lupus erythematosus (SLE) is a severe and chronic autoimmune disorder. Its cause is unknown, but it is believed to result from a complex interaction between genetics and environmental exposures [1, 2]. It affects women more than men and incidence tends to be highest between the ages of 15 and 44 years [3, 4]. The incidence and prevalence of SLE is considerably elevated worldwide among non-white racial groups. For example, the prevalence of SLE among the Aborigine population in Australia was estimated between 13 and 93 per 100,000 population [5].

Currently, there are few incidences and prevalences of SLE studies in Canada. The prevalence of SLE in the First Nations Population of Alberta has been estimated at 27.3 cases and 3.2 cases per 10,000 for females and males, respectively [6]. The prevalence of systemic autoimmune rheumatic diseases (SARDs) in Alberta was 2.6 cases per 1000 residents [1], and the prevalence of SARDs in four Canadian provinces (Manitoba, Quebec, Alberta and Saskatchewan) ranged from 15.9/100,000 in Quebec to 23.0/100,000 in Manitoba [7]. The population-based study conducted to evaluate the prevalence of SLE in First Nations populations, in the province of Manitoba, reported that the prevalence of SLE was twofold (42.3 per 100,000) compared to non-First Nations [8]. Further, the prevalence of SLE British Columbian’s Nuu-Chah-Nulth population was estimated at 0.3%. The Nuu-Chah-Nulth are a tribe of 2300 Pacific First Nations in Canada [9]. The findings of Barnabe and colleagues suggested that the burden of SLE was two times more in females above the age of 45 years than the non-First Nations females [6]. The population-based study in the four provinces of Canada also suggested that SARDs were more common in females than in males across all provinces [7]. Although the study is dated, the annual incidence of SLE in the First Nations populations relative to the general populations has been estimated in Alaskan Indian tribes, the annual incidence of SLE was 9 per 100,000 people [10].

Understanding the incidence and prevalence of SLE may help understand the burden associated with the condition and facilitate resource allocation to improve the quality of life of people with SLE. It may also provide clinicians and policy-makers with valuable information for prioritization of services and estimation of the impacts of policy and practice decisions. While there have been some studies looking at specific aspects of the burden of SLE in Alberta, there is no current systematic approach to monitoring changes in incidence and prevalence of SLE in Alberta. The purpose of the study was to estimate the incidence and prevalence of lupus in the Canadian province of Alberta, using routinely collected administrative health data.

## Methods

The province of Alberta maintains a publicly funded, universally available health care system. Registration with the Alberta Health Care Insurance Plan (AHCIP) is mandatory for all residents of the province. Each resident of the province (approximately 4.2 million) is issued a Personal Health Number (PHN), which acts as a unique lifetime identifier. The PHN is recorded for all contacts with the health care system and, therefore, allows for deterministic linkage across multiple data sources. Ethical clearance and patient written informed consent were not required as this was a retrospective review utilizing population-level administrative health records. All data were anonymized by the Ministry of Health.

## Data sources

### AHCIP central stakeholder registry

The AHCIP central stakeholder registry (CSR) maintains demographic information on all residents of the province eligible for health insurance coverage. This includes date of birth, sex, address, and postal code. Members of the Canadian military and federal inmates are not eligible for provincial health insurance coverage and were excluded from the study.

### Fee-for-service

Most physicians in the province submit claims for reimbursement of health services provided. For those on alternative payment programs, a shadow claim is submitted for services provided. Physicians submitting claims provide information on the type of service provided and record up to three diagnostic codes, using the 9th revision of the International Classification of Diseases (ICD-9), at the 4-digit level. Prior to 1994, only one diagnostic field was required and it used 3-digit ICD-9 coding. Other information includes date and location of services provided, as well as the amount paid to physician.

### Hospital discharge abstract database

Hospital discharge abstract database (DAD) records hospital separations (discharge, transfer, death) and includes up to 25 diagnostic codes. From 2003/2004 fiscal year, the coding was done using the 10th revision of the International Classification of Diseases—Canadian Adaptation (ICD-10-CA). Prior to 2003/2004, the 9th revision of the International Classification of Diseases—Clinical Modification (ICD-9-CM) was in use. The most responsible diagnosis, the one that contributed the most to length of stay, was indicated as well as admission and separation dates, and the type of services provided.

### Case definition

All fee-for-service data were extracted where the ICD-9 code 710.0 (systemic lupus erythematosus) was recorded in any of the three diagnostic fields, for data from 1994 to 2016. For data from 1983 to 1993, ICD-9 code 710 was used. For inpatient data, all records with either ICD-9-CM code 710.0 or ICD-10-CA code M32.xx recorded were extracted. The period 1983–1994 was used as a run-in period to separate incident and prevalent cases. A case was defined as any individual that had at least three physician services, over a 2-year period, with a minimum of 60 days between the first and second services, or one or more hospitalizations.

### Data analysis

Age-standardized and age- and sex-specific incidence and prevalence estimates were computed, using the AHCIP CSR mid-year population estimates as the denominator. Incidence and prevalence estimates were age standardized, using the direct method, to the 1991 Canadian census population. Confidence intervals were calculated using the method developed by Carriere and Roos [11].

## Results

### Prevalence

A total of 1442 cases were identified as having new or existing SLE for all age groups in Alberta for the year 2000. This is equivalent to an overall prevalence of 47.99/100,000. From this, the proportion of female (*n* = 1,237) and male (*n* = 205) was 83.2/100,000 and 13.5/100,000, respectively (see Table 1).

**Table 1:** The prevalence of SLE for the year 2000 in Alberta, Canada (per 100,000)

Sex	Total population	No. of cases	Prevalence
Female	14,867,74	1237	83.20
Male	15,174,24	205	13.51
Total	30,041,98	1442	48.0

In the year 2000, a prevalence of 112.46/100,000 and 17.88/100,000 of SLE was identified in female and male over 19 years of age, respectively, in Alberta. On the other hand, a proportion of 2.74/100,000 in male and 7.48/100,000 in female was identified with SLE under the age of 19 years (see Table 2).

**Table 2:** The prevalence of SLE in childhood vs adult for the year 2000 in Alberta, Canada (per 100,000)

	Total population	No new case	Prevalence
Female < 19 years	414,374	31	7.48
Male < 19 years	437,816	12	2.74
Female > 19 years	10,724,00	1206	112.46
Male > 19 years	10,796,08	193	17.88

**Fig. 1:**
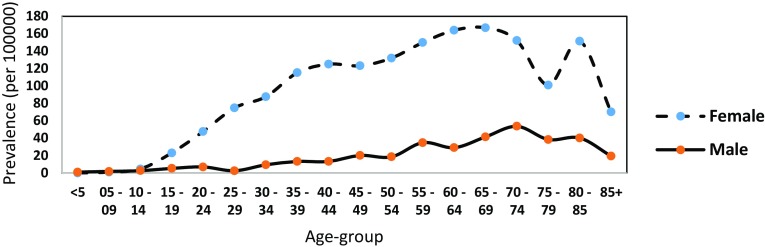
The prevalence of SLE for male and female by age for the year 2000

The peak prevalence for male = 53.59/100,000 and female = 166.8/100,000 was estimated between the ages of 70–74 and 65–69, respectively, for the year 2000 (see Fig. 1). For girls under the ages of five, one patient with SLE was identified out of 99,328 which is equivalent to the point prevalence of 1/100,000. On the other hand, no case of SLE was identified out of 94,168 suspected SLE male patients under the ages of five. Generally, the prevalence for both male and female was observed to increase with age.

**Fig. 2:**
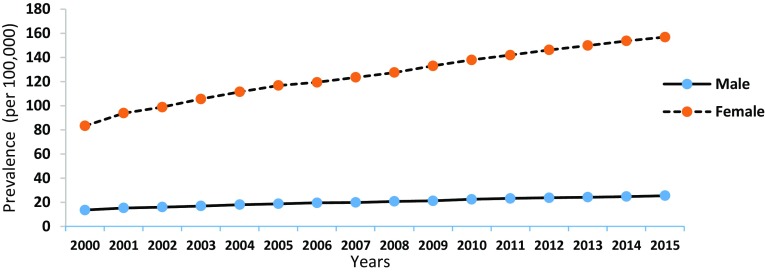
The prevalence of SLE for male and female over 16 years (2000–2015)

The trend of SLE prevalence between 2000 and 2015 is presented below (see Fig. 2). The proportion of people with SLE has increased from 1442 (Population = 3,002,917) to 3773 cases (Population = 4,193,964) between 2000 and 2015, respectively. In other words, in 16 years time, the prevalence of SLE has increased by 42 (male = 12, female = 73.5) per 100,000 population.

### Incidence

**Fig. 3:**
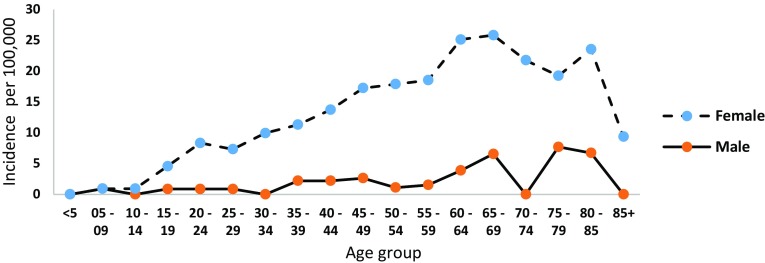
The incidence of SLE for male and female by age for the year 2000

The overall incidence of SLE between 2000 and 2015 was 4.43 (95% CI 3.65, 5.04) per 100,000 population. From this, the overall incidence of male and female was 1.3 (95% CI 0.72, 1.76) and 7.69 (95% CI 6.22, 8.81) per 100,000 population, respectively (see Table 3). In the year 2000, there were no new cases of SLE patients for both male and female who are less than 5 years of age (see Fig. 3). The peak incidence of SLE was recorded for male (7.68/100,000) and female (21.75/100,000) patients aged 75–79 and 70–74, respectively.

**Table 3:** The overall incidence of SLE by sex (per 100,000)

Sex	Population	No. of new cases	Incidence
Female	1,749,741	133.4	7.60 (6.22, 8.81)
Male	1,803,354	22.6	1.30 (0.72, 1.76)
Total	3,553,095	156	4.42 (3.65, 5.04)

**Fig. 4:**
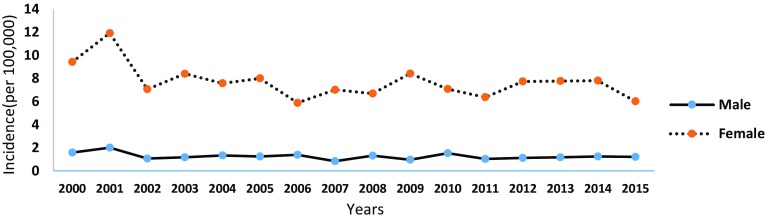
The incidence of SLE for male and female over 16 years (2000–2015)

The trend of SLE incidence between 2000 and 2015 is presented below (see Fig. 4). Compared to the year 2000, the incidence of SLE for both sexes in 2015 has decreased by 1.85 (male = 0.36, female = 3.4) per 100,000 population. The peak incidence of SLE (6.8/100,000) was recorded in the year 2001 for both male and female. The incidence of SLE in female between the years of 2000 and 2015 was approximately six times greater than their male counterpart.

The number of new cases of SLE in 2012, 2013, 2014 and 2015 was 4.37 (male = 1.11, female = 7.73), 4.42 (male = 1.17, female = 7.77), 4.47 (male = 1.24, female = 7.81) and 3.58 (male = 1.22, female = 6.01) per 100,000 population, respectively (see Table 4). The average incidence difference between the consecutive years was not substantial, and this implies that the incidence of SLE over the 4-year period was approximately stable.

**Table 4:** The incidence of SLE in Alberta, Canada (per 100,000)

	2012	2013	2014	2015
Total	4.37	4.42	4.47	3.58
Female	7.73	7.77	7.81	6.01
Male	1.11	1.17	1.24	1.22

## Discussion

The aim of the study was to estimate the incidence and prevalence of SLE in Alberta, Canada. It used multiple population-based data sources including the Alberta Health Care Insurance Plan Central Stakeholder Registry (AHCIP CSR), Fee-For-Service, and Hospital Discharge Abstract Database. A prevalence of 47.99 per 100,000 (male = 13.5, female = 83.2) of SLE was observed for the year 2000, and has increased to 90 (male = 25.5, female = 156.7) per 100,000 population in 2015. This study was compared with a population-based study conducted in the First Nations populations of Alberta [6]. We observed the prevalence of 2.73 cases per 100,000 female and 0.32 cases per 100,000 male in the First Nations populations [6]. The prevalence rate of SLE in the First Nations of Population was lower than the current study. This may be because the prevalence of SLE was estimated only from 6% of the Alberta populations who were ≥ 45 years. Approximately, a similar prevalence of SLE (42.3/100,000) to the current study was also reported in the Firth Nations population in Canada [8].

The prevalence in the current study was compared to studies conducted in United Kingdom (UK) [12] and the United States of America (USA) [13]. The data sources used for the Birmingham, UK study were the lupus patient support group, and hospital inpatient and laboratory data and notification by attending and the primary care physician. The prevalence rate of SLE (27.7/100,000) in Birmingham was approximately half of the prevalence rate in Alberta, Canada. On the other hand, the prevalence rates of SLE in California (107.6/100,000) and Pennsylvania (149.5/100,000) were two times higher than this current study [13]. The cause of the dramatic differences in prevalence of SLE between countries may be due to the methodology issues such as the size and type of sample used for data collection. For example, the current study was based on population of all age groups; this is because patients in Canada have no financial barriers to accessing healthcare, as they have in the United States. In addition, the environmental factors such as drugs, physical or mental stress, and air pollution may contribute to the high number of people with SLE in some countries. For example, the potential cause of SLE cases in Alberta could be air pollution, nitrogen dioxide and systemic autoimmune rheumatic disease [1].

This study is the first of its kind in Alberta to report the overall incidence rate of SLE using a population-based data collected between 2000 and 2015. The overall incidence of SLE for all ages in Alberta was 5.39/100,000. This observed incidence rate of SLE was compared to the most recent studies carried out in Spain [14] and Iceland [15]. The incidence rate of SLE (2.15/100,000) in Spain was lower than in Alberta. Similar to this, the incidence rate of SLE in Iceland was estimated at 3.3/100,000, again this is lower than the present study. The possible factor for the differences in the incidence estimate between these studies could be the availability of cases of SLE to be used as a data source. For example, some milder cases of SLE may never present to hospital or be just treated as anaemia with skin rash by physicians.

Observation of the peak incidence rate of SLE in the current study for both female (21.75/100,000) and male (7.68/100,000) at the ages of 70–74, and 75–79 was made, respectively. Contrary to our study, the peak incidence rate of SLE in females in Birmingham, UK was reported between the ages of 18 and 19 years and a similar finding in the USA suggested the peak incidence rate of SLE was between the ages of 15 and 44 [12, 16]. However, studies from Nottingham, Sweden and Iceland have reported that the peak incidence rate was similar to that observed in current study which was between the ages of 40–49, 40–50 and 55–74, respectively [15, 17, 18]. Reasons for discrepancies in reported peak incidence of SLE in different countries, could be explained by patient characteristics (age, race and socioeconomic) they consider in their study.

There are some strengths and limitations to this study. The strengths to this study include the data being population based, recall bias was not an issue, any misclassification errors on the side of providing conservative estimates, and it is the first of its kind to estimate the incidence of SLE in Alberta. One of the limitations of the study is that our prevalence and incidence estimate were based on use of health services; the impact of this is that we may have misclassified or estimated cases of SLE. This study was also limited in taking account of the geographic distribution of cases. Further, we do not have any data on deaths attributable to SLE, as a result this could not be included. Individuals that died, for any reason, were removed from prevalence estimates. Future research may include looking at the geographic distribution of cases to better assess potential environmental influences, direct health care costs, and pharmaceutical use.

## Conclusion

This study has estimated the prevalence and incidence of SLE in Alberta based on multiple population-based data sources. It is the first of its kind to estimate for both male and female in all age groups of population. Despite the stable incidence of SLE, the present study confirms that the prevalence of SLE has increased over the 16-year period. The increase in prevalence of SLE in Alberta will have an impact on health service utilizations. This finding can, therefore, be used for planning and evaluating health services for this group of patients. Future research may include looking at geographic distribution of cases, direct health care costs, and pharmaceutical use of SLE.
